# Automatic grading of HER2 immunohistochemical whole-slide images based on a multi-scale hierarchical graph neural network

**DOI:** 10.3389/fpubh.2026.1940487

**Published:** 2026-09-29

**Authors:** Yingjie Jiang, Wenhao Sun, Chao Ye, Chun Xu, Jiewen Xue, Yiliang Wang, Shiju Yan, Yiming Li, Chenguang Bai

**Affiliations:** 1 Department of Pathology, Changhai Hospital, Naval Medical University, Shanghai, China; 2 School of Health Science and Engineering, University of Shanghai for Science and Technology, Shanghai, China; 3 The 902nd Hospital of the Joint Logistic Support Force, Bengbu, China; 4 CellSee(Wuxi) Intelligent Technology Co., Ltd, Wuxi, China; 5 School of Basic Medical Sciences, Naval Medical University, Shanghai, China

**Keywords:** artificial intelligence, automated HER2 grading, breast cancer, graph neural network, HER2 immunohistochemistry, whole-slide imaging

## Abstract

**Objective:**

The HER2 immunohistochemical (IHC) score of breast cancer is a critical precursor for determining eligibility for anti-HER2 targeted therapy, as the final HER2 status (positive/negative/low) is determined by IHC results combined with FISH confirmation for equivocal 2 + cases according to ASCO/CAP guidelines. However, most existing artificial intelligence methods separate the detection of invasive cancer regions from HER2 grading and fail to adequately model the spatial heterogeneity of whole-slide tumors. This study proposes an end-to-end intelligent HER2 grading framework that integrates automated detection of invasive cancer regions with hierarchical graph reasoning, enabling automated and precise evaluation of HER2 IHC in breast cancer.

**Method:**

A coarse-to-fine automated HER2 grading framework was developed. First, invasive tumor regions were automatically identified through supervised multi-scale tissue segmentation. Within the detected regions, patch-level HER2 grading integrates Delaunay cell image features with self-supervised visual representations, while WSI-level spatial representations model tumor heterogeneity to achieve fully automated HER2 grading across whole-slide images. The framework was trained using data from two scanning platforms and externally validated on 82 independent WSI samples from a third platform.

**Results:**

In independent external validation, the Dice coefficient for invasive region detection reached 0.89 (on 50 WSI images); the accuracy rate of HER2 grading at the patch level was 96.49% (95% CI, 96.08–96.87%) (7,924 patches), while that at the WSI level reached 98.0% (95% CI, 91.5–99.9%) (82 WSI images). The quadratic-weighted Cohen’s κ was 0.97 (95% CI, 0.94–1.00), and all misclassifications at the WSI level were confined to the distinction between HER2 1 + and 2 +.

**Conclusion:**

This framework enables fully automated HER2 IHC scoring and interpretation under multicenter and cross-scanner conditions, providing a computational tool for standardized IHC assessment, while final HER2 status determination for 2 + cases still requires FISH validation.

## 1 Introduction

HER2 (human epidermal growth factor receptor 2, encoded by the ERBB2 gene) is a critical molecular target for precision therapy in breast cancer, with approximately 15–20% of breast cancers exhibiting HER2 amplification or protein overexpression (1, 2). The rapid development of anti-HER2 therapies, particularly trastuzumab and the antibody–drug conjugate trastuzumab deruxtecan (T-DXd), has markedly improved clinical outcomes, making accurate HER2 assessment essential for therapeutic decision-making and prognostic evaluation (3). Furthermore, the emergence of the HER2-low concept has shifted HER2 assessment from a simple binary classification toward a more refined evaluation of HER2 expression, placing increasingly stringent demands on the accuracy, reproducibility, and standardization of HER2 immunohistochemistry (IHC) interpretation (4).

Currently, HER2 expression is initially assessed by immunohistochemistry (IHC) according to the 2023 American Society of Clinical Oncology/College of American Pathologists (ASCO/CAP) guideline, which classifies staining into four categories (0, 1+, 2+, and 3+). However, it is critical to note that IHC 2 + is an equivocal result requiring reflex FISH testing for final HER2 status determination, while only IHC 3 + is directly considered HER2-positive and IHC 0/1 + as negative or low-expression (5). However, HER2 IHC interpretation remains highly dependent on pathologists’ experience, particularly for low-expression and equivocal cases (1 + and 2+), resulting in considerable interobserver variability even among experienced breast pathologists (6). Although recent advances in artificial intelligence (AI) have substantially improved automated HER2 assessment, comparative studies have demonstrated only moderate agreement among different AI models, indicating that further improvements in robustness and generalizability are still required (7). Therefore, developing accurate, reliable, and scalable AI-assisted HER2 grading methods remains of considerable clinical significance.

In recent years, deep learning has substantially advanced automated HER2 immunohistochemistry (IHC) grading from patch-level classification to whole-slide image (WSI)-level assessment. Early studies primarily combined convolutional neural networks with handcrafted membrane staining features, such as staining intensity, membrane completeness, and the proportion of positive tumor cells, but did not explicitly model cellular spatial relationships (8). Subsequently, multi-stage deep learning frameworks incorporating additional Calponin immunohistochemistry-stained serial sections improved the identification of invasive carcinoma regions and HER2 scoring consistency, although the requirement for additional staining increased both cost and registration complexity (9).

More recently, pathology foundation models based on Transformer architectures have further improved WSI-level HER2 scoring performance; however, these approaches mainly rely on visual representation learning without explicitly modeling HER2 membrane morphology or pathological interpretation rules (10). Graph neural networks have also been introduced into computational pathology to capture spatial relationships between cells and tissues. For example, SlideGraph+ employed Delaunay cell graphs for HER2 status prediction, demonstrating the value of topological modeling, although it was developed on H&E slides rather than HER2 IHC images (11). In addition, multiscale sampling strategies and deep neural network–based classification models have further promoted WSI-level HER2 scoring (12, 13). Nevertheless, most existing methods still rely on patch voting or simple probability aggregation, without jointly modeling membrane staining morphology, cellular spatial topology, and WSI-level spatial heterogeneity.

Based on current research, automated HER2 immunohistochemistry (IHC) grading still faces three major challenges. First, existing methods mainly rely on visual representations while insufficiently integrating HER2 membrane morphological characteristics with cellular spatial topology. Second, invasive carcinoma detection and HER2 grading are usually performed as separate tasks, and some approaches require additional immunostaining, increasing workflow complexity and limiting clinical applicability. Third, whole-slide image (WSI)-level prediction is commonly based on patch voting or simple probability aggregation, which inadequately captures the spatial heterogeneity of tumor tissues. Therefore, developing an accurate, interpretable, and clinically applicable HER2 grading framework that jointly models membrane morphology, cellular organization, and WSI-level spatial context remains an important challenge.

To address these challenges, we propose an end-to-end HER2 grading framework integrating automatic invasive carcinoma detection with hierarchical graph learning. Specifically, the proposed framework combines self-supervised visual representations and Delaunay cell graphs to jointly characterize membrane staining morphology and cellular spatial topology, and further incorporates WSI-level graph learning to model tissue spatial heterogeneity. The main contributions of this study are threefold: (1) an automatic invasive carcinoma detection method without additional immunostaining; (2) a graph-based HER2 grading model integrating membrane morphological features with cellular spatial topology; and (3) a WSI-level graph inference strategy incorporating spatial consistency to improve grading robustness and generalizability.

## 2 Materials and methods

### 2.1 Materials

#### 2.1.1 Research design and ethical review

This study was a multicenter retrospective investigation into diagnostic accuracy, conducted in compliance with the STARD 2015 (Standards for Reporting Diagnostic Accuracy Studies) reporting guidelines (14). The study protocol was approved by the Ethics Committee of Changhai Hospital (Approval No. CHEC2022-109, approved on October 9, 2023). All WSI data were anonymized by removing personal identification information such as patient names and medical record numbers, retaining only study codes. As the study employed previously archived pathological sections for retrospective analysis, the ethics committee waived the requirement for patient informed consent.

The study included consecutive cases collected from three institutions between January 2018 and December 2023. Inclusion criteria were: (1) female patients aged ≥18 years; (2) histopathologically diagnosed with invasive breast carcinoma of no special type (IBC-NST); (3) availability of surgical resection specimens with HER2 immunohistochemical (IHC) staining; (4) absence of prior neoadjuvant or anti-HER2 therapy. Exclusion criteria included: (1) specimens from recurrent or metastatic breast cancer; (2) puncture biopsy specimens; (3) sections with significant tissue or staining artifacts affecting image analysis quality. A total of 482 patients were ultimately included in the analysis. The overall cohort had a median age of 56 years (interquartile range, 49–64 years), with median ages of 56 years, 57 years, and 55 years in Changhai Hospital, the 902nd Hospital, and Naval Medical University, respectively. All included cases were histopathologically confirmed invasive breast carcinoma of no special type (IBC-NST). The HER2 IHC distribution was 47 cases with score 0, 104 with score 1+, 186 with score 2+, and 145 with score 3+; the center-specific distributions are summarized in Table 1.

**Table 1:** Data sources and scoring distribution of HER2 IHC WSI results across three hospitals.

Institution	Abbreviation	Scanner	Resolution (μm/pixel)	Age, median (IQR)	WSIs	0	1+	2+	3+
Changhai Hospital	DPCH	Aperio GT450 DX	0.235	56 (49–64)	300	28	56	129	87
902nd Hospital	902nd	KFbio EX KF pro 005EX	0.241	57 (50–65)	100	10	20	38	32
Naval Medical University	SBMS	Leica Aperio AT2	0.208	55 (48–63)	82	9	28	19	26
Total	–	–	–	56 (49–64)	482	47	104	186	145

#### 2.1.2 Whole slide image acquisition and quality control

The HER2 IHC-stained tissue sections from the three institutions were fully digitized using three different models of digital pathology scanners to evaluate the model’s cross-platform generalization capability. Changhai Hospital employed an Aperio GT450 DX scanner (Leica Biosystems, Germany) with a scanning resolution of 0.235 μm/pixel, generating a total of 300 WSI images; 902nd Hospital utilized a KFbio EX Magscanner KF pro 005EX scanner (Ningbo Jiangfeng Bioinformatics Technology Co., Ltd., China) with a scanning resolution of 0.241 μm/pixel, producing 100 WSI images; Naval Medical University employed an Aperio AT2 scanner (Leica Biosystems, Germany) with a scanning resolution of 0.208 μm/pixel, obtaining 82 WSI images.

Representative WSIs are presented in Supplementary Figure S1, showing the same tumor field of view (100×) for each HER2 grade across the three scanners. The images reveal scanner-specific differences in color, optical density, and noise level. The Aperio GT450 DX exhibits the highest DAB optical density with a warm-toned appearance, but strong positive regions are prone to pixel saturation; the Aperio AT2 shows higher hematoxylin gain with a cold-toned characteristic and suppressed DAB signals; the KFbio EX displays relatively neutral color but with higher background stromal noise. These differences generally do not interfere with pathologists’ visual interpretation but may introduce scanner- and institution-specific variations that pose challenges for pixel-intensity-based AI algorithms, including signal saturation, intensity underestimation, and elevated false-positive rates in weakly stained regions. In addition, the scanners differed in spatial resolution (0.235, 0.241, and 0.208 μm/pixel, respectively), further contributing to heterogeneity in image characteristics across scanning platforms.

Although color normalization, stain normalization, and resolution harmonization can reduce low-level imaging variability, they cannot fully eliminate scanner-specific differences in optical response, dynamic range, image noise, or institution-specific staining characteristics. Therefore, independent cross-center validation remains necessary to assess model robustness under real-world domain shifts. Accordingly, independent multicenter and cross-scanner validation was performed to evaluate the robustness and generalizability of the proposed framework under these heterogeneous imaging conditions.

All WSI images underwent a standardized quality control process, including image integrity checks, tissue region confirmation, and staining quality assessment. Sections exhibiting severe tissue folding, tearing, uneven staining, or scanning artifacts were excluded. Ultimately, 482 high-quality WSI images were selected for study analysis, with training, validation, and external testing performed on non-overlapping WSI cohorts.

#### 2.1.3 Pathological annotation and dataset construction

All WSI-grade HER2 scores were independently and double-blindly determined by two pathologists with experience in breast pathology diagnosis according to the 2023 ASCO/CAP HER2 testing guidelines. For cases with inconsistent scores, a third senior breast pathology expert conducted a review to establish the final classification. The consistency of dual-pathologist WSI-grade scoring was assessed using a two-way weighted Cohen’s κ, which demonstrated high agreement (κ = 0.93; 95% CI: 0.91–0.95) (15). The reference standard for model development and evaluation was the final expert-assigned HER2 IHC score according to the 2023 ASCO/CAP guideline. Because FISH confirmation was not available for all HER2 2 + equivocal cases, the present model should be interpreted primarily as an automated IHC grading system rather than a direct predictor of molecularly confirmed HER2 amplification status.

The invasive carcinoma region detection dataset comprises 150 WSI images (100 from Changhai Hospital and 50 from the 902nd Hospital) (Table 2), with boundaries of invasive carcinoma regions independently annotated by two pathologists and quality-controlled using the Dice similarity coefficient. Regions with inconsistent annotations were revised by a third pathology expert, resulting in 150 high-quality annotated WSI images for training and testing of the invasive region detection model. The average annotation consistency Dice coefficient was 0.94 (range: 0.89–0.98).

**Table 2:** Detailed segmentation of the HER2 grading dataset and distribution of patch-level annotations.

Dataset	Institution	WSIs	Patches	Patch distribution by HER2 score				Purpose
				0	1+	2+	3+	
Self-supervised pretraining	DPCH	150	238,089	–	–	–	–	DINOv2 representation learning
	902nd	50	78,807	–	–	–	–	DINOv2 representation learning
Subtotal	DPCH+ 902nd	200	316,896	–	**–**	**–**	**–**	
Training	DPCH	40	3,870	362	1,370	1,078	1,060	Model optimization
	902nd	40	3,784	358	1,278	998	1,150	Model optimization
Subtotal	DPCH+ 902nd	80	7,654	720	2,648	2,076	2,210	
Validation	DPCH	10	995	93	360	280	262	Hyperparameter tuning / Early stopping
	902nd	10	968	92	332	250	294	Hyperparameter tuning / Early stopping
Subtotal	DPCH+ 902nd	20	1,963	185	692	530	556	
Test	SBMS	82	7,924	725	2,902	1,901	2,396	Independent external evaluation

The patch-level HER2 classification annotation was independently performed by two pathologists, who classified patches into four grades (0, 1+, 2+, and 3+) according to ASCO/CAP standards. Discrepant patches were adjudicated by a third expert. A total of 17,541 annotated patches were ultimately obtained for supervised learning, with a Cohen’s κ for inter-physician annotation consistency of 0.91 (95% CI: 0.87–0.92).

The dataset partitioning for the HER2 grading task is detailed in Table 2. To prevent data leakage, the invasive region detection task and the HER2 grading task utilize separate datasets. During the self-supervised pre-training phase, a total of 200 WSI images from Changhai Hospital and 902nd Hospital were used; after automatic identification of invasive regions, 316,896 tissue patches were extracted for DINOv2 feature learning (16). The supervised HER2 grading model employed 100 WSI images from both institutions, comprising 80 images for training (7,654 patches) and 20 images for validation (1,963 patches). Additionally, 82 WSI images from Naval Medical University served as an independent external test set, containing 7,924 patches. Detailed data distributions are presented in Tables 1–3. All dataset partitions were performed at the patient/WSI level, and the pretraining, supervised training, validation, and external test cohorts contained non-overlapping patients and WSIs. The 82 WSIs from Naval Medical University were reserved exclusively for independent external evaluation and were not used for model selection, hyperparameter tuning, early stopping, threshold selection, feature selection, or any other model-development decision.

**Table 3:** Division of the regional detection dataset for invasive carcinoma.

Dataset	Institution	WSIs	WSI distribution byHER2 score				Annotation
			0	1+	2+	3+	
Training	DPCH	100	10	35	20	35	Manual invasive tumor region annotation
External test	902nd	50	5	18	10	17	Manual invasive tumor region annotation
Total	DPCH + 902nd	150	15	53	30	52	

### 2.2 Methods

The proposed framework consists of six stages that span from image acquisition to final HER2 score generation. An overview of the complete workflow is presented in Figure 1. Briefly, HER2-stained WSIs are first digitized and undergo automated invasive carcinoma region detection using multi-scale tissue representations extracted by Virchow2 and segmented by SAM2, restricting all subsequent analysis to clinically relevant invasive tumor areas (Section 2.2.1). Within the detected regions, cell nuclei and membranes are segmented to construct Delaunay cell graphs for learning cell-level morphological and spatial representations, while DINOv2 extracts self-supervised visual features from tissue patches. The graph and visual features are fused for patch-level HER2 prediction, and all patch representations are further integrated through hierarchical graph learning to model WSI-level spatial heterogeneity and generate the final HER2 score (Section 2.2.2).

**Figure 1:**
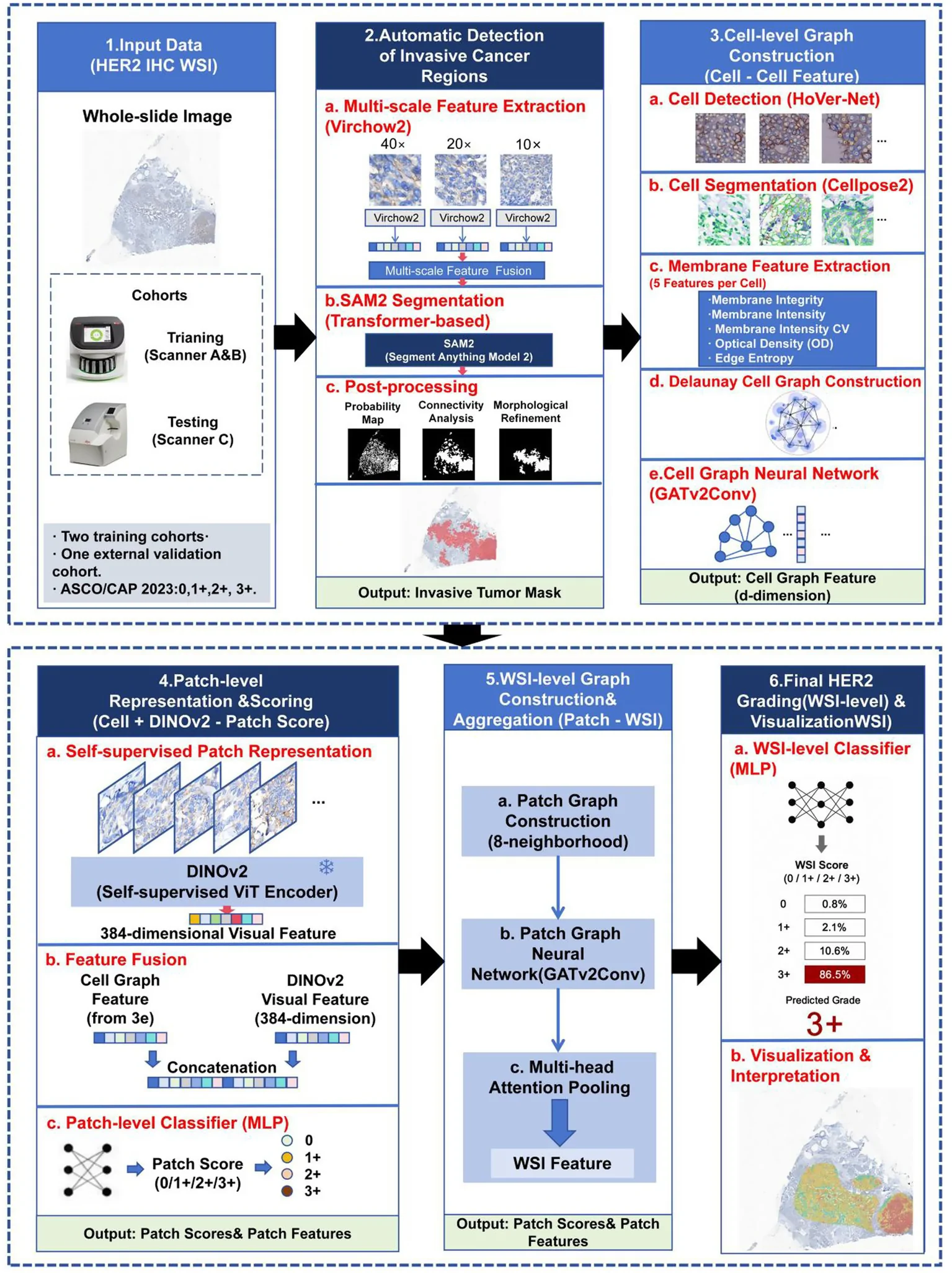
Overall framework of the proposed multi-scale hierarchical graph learning framework for automatic HER2 immunohistochemistry (IHC) whole-slide image (WSI) grading. The framework consists of six stages: (1) HER2 WSI acquisition; (2) Automatic invasive carcinoma region detection using multi-scale tissue representations extracted by Virchow2 and segmented by SAM2, restricting subsequent analysis to clinically relevant invasive tumor areas; (3) Nuclei and membrane segmentation followed by Delaunay cell graph construction for learning cell-level morphological and spatial representations; (4) DINOv2 self-supervised visual feature extraction to generate a 384-dimensional feature representation, which is fused with cell graph representations for patch-level HER2 prediction; (5) Hierarchical graph learning to aggregate patch representations into a WSI-level representation while modeling spatial heterogeneity; and (6) WSI-level HER2 prediction with interpretable probability maps and heatmap visualization.

#### 2.2.1 Automatic detection of invasive cancer regions

According to the ASCO/CAP2023 HER2 testing guidelines, the HER2 IHC score should be assessed exclusively within the invasive tumor region. Therefore, this study first developed an automated detection model for identifying invasive cancer regions in WSI images, and utilized these predicted regions for subsequent HER2 grading analysis.

Specifically, manually annotated invasive cancer regions were used as supervised labels to construct a WSI-level region segmentation dataset. First, tissue image features were extracted using the Virchow2 foundation model (17) at magnifications of 40×, 20×, and 10×, followed by spatial alignment and multi-scale feature fusion to obtain WSI representations that capture information across different tissue scales. The combined features were then fed into a Transformer-based segmentation network (SAM2) (18), which learns the correspondence between tissue structural features and invasive cancer regions to achieve pixel-level region prediction.

Model training utilized manually annotated masks of invasive cancer regions as supervision signals, employing both Dice Loss and Binary Cross-Entropy Loss to optimize model parameters and enhance tumor boundary recognition accuracy. During inference, the model-generated probability maps were used to generate invasive cancer region masks, followed by connectivity analysis and morphological post-processing to eliminate isolated noise areas, yielding final invasive cancer boundaries. Subsequent HER2 patch-level and WSI-level analyses were conducted exclusively within these automatically identified regions. During inference, the probability map generated by SAM2 was converted to a binary mask using a threshold of 0.5. Connected components smaller than 1,000 pixels were removed, followed by morphological opening and closing using a 5 × 5 binary kernel to suppress isolated noise and improve boundary continuity.

#### 2.2.2 Construction of the HER2 intelligent grading framework based on hierarchical graph reasoning

Given that HER2 expression exhibits both membrane staining heterogeneity at the cellular level and spatial distribution characteristics at the tissue level, this study establishes a three-tiered hierarchical graph reasoning framework to enable multi-scale HER2 analysis spanning from cells to patches to WSI.

First, to enhance tissue representation capabilities with limited labeled data, we employed the DINOv2 self-supervised learning strategy for pre-training on 316,896 unlabeled patches extracted from 200 WSI images. After training, the encoder parameters were frozen, and the model was used to extract 384-dimensional patch-level visual features that capture comprehensive tissue morphology information.

For the 512 × 512 patches within invasive cancer regions, HoVer-Net was first employed to perform cell nuclei segmentation and classification (19), and cell type identification, followed by Cellpose 2 to determine epithelial cell membrane boundaries (20). Based on the membrane segmentation results, five HER2-related membrane staining features were extracted—including membrane integrity, normalized staining intensity, staining intensity coefficient of variation, optical density, and edge angle entropy—to characterize HER2 membrane expression patterns. For each epithelial cell, five membrane staining features were calculated from the segmented membrane region. Membrane integrity was computationally approximated as the proportion of the membrane contour covered continuously by the segmented membrane mask with positive DAB staining. Normalized staining intensity was calculated as the mean normalized DAB intensity within the membrane region. The staining intensity coefficient of variation was calculated as the standard deviation divided by the mean normalized membrane intensity. Optical density was calculated as OD = −log10(I/I0), where I denotes the measured intensity and I0 represents the reference background intensity. Edge angle entropy was calculated using the Shannon entropy of the local membrane edge-orientation distribution.

Subsequently, a Cell Graph was constructed using the cytoplasmic centers of tumor epithelial cells as nodes through Delaunay triangulation, with GATv2Conv (21) employed to learn intercellular spatial topological relationships. Cellular membrane staining features were mapped via MLP to generate continuous HER2 scores, which were then fused with cell spatial representations learned by graph neural networks. Attention pooling was applied to derive Patch-level cellular graph features. These features were further combined with DINOv2 visual features and fed into a Patch-level classifier to achieve four-class classification of HER2 IHC scores: 0, 1+, 2+, and 3 + .

At the WSI scale, to further model spatial correlations between different tissue regions, patches are treated as graph nodes with 8-neighborhood connections defined by spatial positions, forming a WSI-level Patch Graph. Information is propagated between neighboring patches using GATv2Conv, followed by multi-head self-attention pooling to generate a global WSI-level representation for final HER2 classification.

Model training employs a combined patch-level and WSI-level supervised learning strategy, with model parameters optimized using weighted cross-entropy loss. During inference, analysis is limited to infiltrative cancer regions identified by automated detection; Patch weights are calculated based on prediction confidence scores and cellular HER2 scores, enabling weighted fusion of Patch predictions. Additionally, Patch Graph spatial consistency constraints are applied to reduce local prediction noise and enhance WSI-level score stability. The uncertainty threshold was selected using the validation cohort. Candidate top-two probability ratios ranging from 0.70 to 0.95 at 0.05 intervals were evaluated, and 0.85 provided the best balance between classification accuracy and identification of borderline cases. The selected threshold was fixed before external evaluation. Cases with a ratio of the second-highest to the highest category prediction score above 0.85 were classified as “borderline uncertain” and flagged for further review and potential FISH confirmation.

For reproducibility, the supervised models were optimized using AdamW with an initial learning rate of 1 × 10^-4, weight decay of 1 × 10^-4, a batch size of 32, a maximum of 100 training epochs, cosine learning-rate decay, and early stopping with a patience of 15 epochs. The 512 × 512 patches were extracted with a stride of 256 pixels. During training, random horizontal and vertical flipping, random rotation between 0° and 180°, random brightness and contrast perturbation of up to 15%, and Gaussian noise augmentation with a standard deviation of 0.01 were applied. The GATv2Conv module used eight attention heads with a 256-dimensional hidden representation. The computational environment comprised PyTorch 2.2.1, CUDA 12.1, Python 3.10, Ubuntu 22.04, and an NVIDIA RTX 4090 GPU with 24 GB memory.

### 2.3 Statistical analysis

Statistical analyses were performed using Python 3.10. Continuous variables are presented as mean ± standard deviation or median with interquartile range, as appropriate. Ninety-five percent confidence intervals for WSI-level accuracy and quadratic-weighted Cohen’s κ were estimated using 2,000 bootstrap resamples at the patient/WSI level. For patch-level performance metrics, bootstrap resampling was performed at the WSI level to preserve within-WSI clustering of patches. One-versus-rest AUCs and their 95% confidence intervals were estimated using WSI-clustered bootstrap resampling. A two-sided *p* < 0.05 was considered statistically significant.

## 3 Results

### 3.1 Performance of automatic regional detection for invasive carcinoma

To evaluate the performance of automated detection of invasive cancer regions, this study trained the model on 100 WSI images from Changhai Hospital and conducted external validation on 50 independent WSI images from the 902nd Hospital. Using pathologist-annotated invasive cancer regions as the reference standard, the model achieved a Dice coefficient of 0.89 (95% CI, 0.86–0.92) on the external test set.

As shown in Figure 2, the model effectively identifies infiltrative cancer regions across varying HER2 expression levels and tissue contexts while minimizing interference from normal ductal structures, adipose tissue, and stromal areas, thereby providing a standardized analysis region for subsequent automated HER2 scoring. The nuclear-density clustering in Figure 2D was included as an interpretability analysis rather than an independent segmentation metric. Across the 50 external test WSIs, high-density tissue clusters showed a mean overlap of 0.81 ± 0.07 with pathologist-annotated invasive carcinoma regions, supporting the relevance of nuclear-density variation as a structural cue for invasive-region localization.

**Figure 2:**
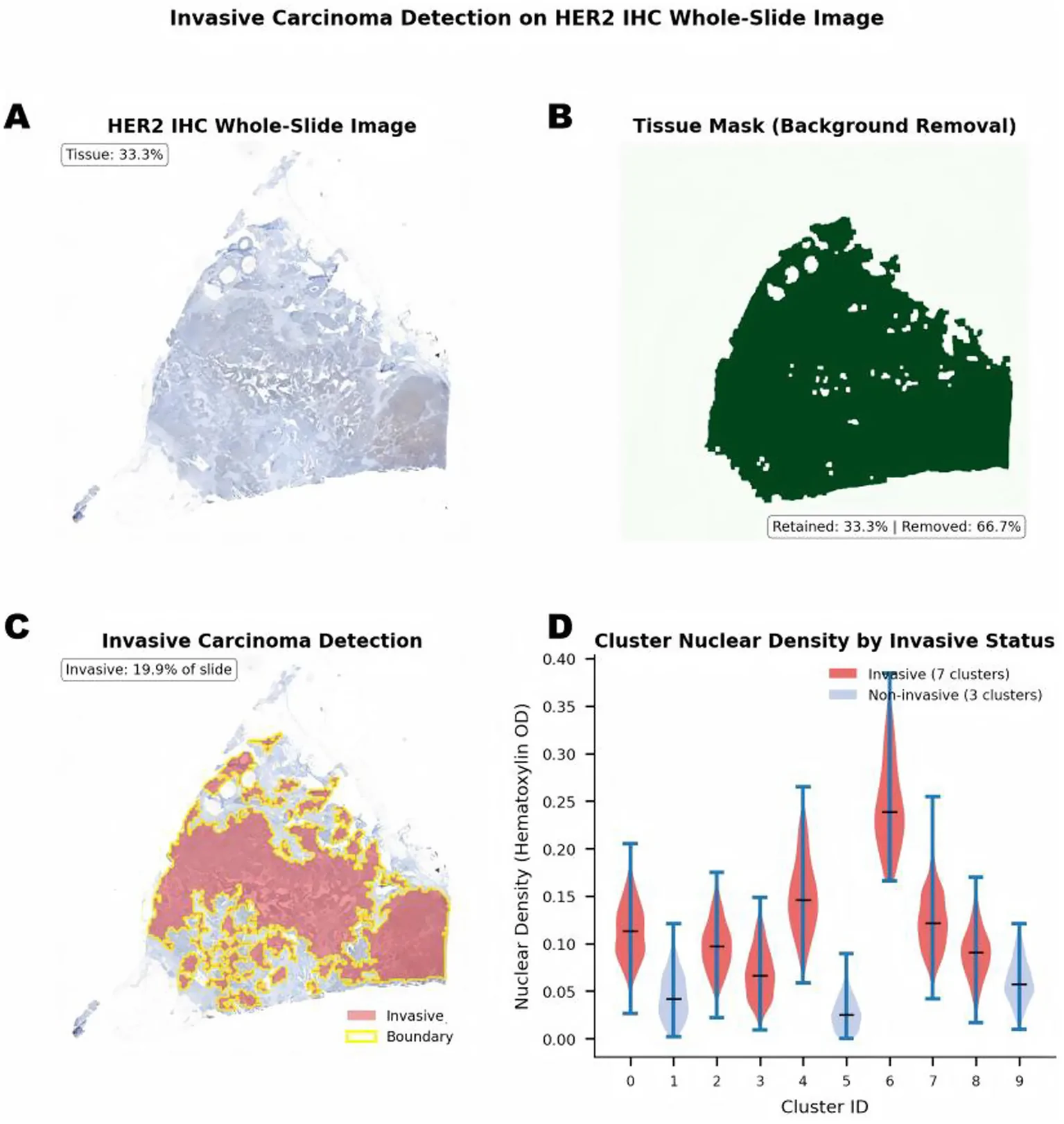
Representative imaging characteristics and automated invasive carcinoma region detection. **(A)** Representative HER2 IHC WSIs from Changhai Hospital, the 902nd Hospital, and Naval Medical University, illustrating inter-center differences in staining appearance and image acquisition characteristics. **(B)** Tissue region extraction via Otsu thresholding (green, retained tissue; white, background). **(C)** Automated invasive carcinoma detection overlay (red, predicted invasive regions; yellow contours, boundaries). Normal ducts, adipose tissue, and stromal areas are effectively excluded. **(D)** Nuclear density distribution by tissue cluster (hematoxylin optical density), with high-density clusters corresponding predominantly to invasive carcinoma and low-density clusters to non-invasive tissue.

### 3.2 Performance of patch-level HER2 classification

In our independent external test set for the third scanning platform of School of Basic Medical Sciences (SBMS) (Leica Aperio AT2), we conducted patch-level evaluations on 82 untrained WSI images, identifying a total of 7,924 patches corresponding to invasive tumors.

The model achieved an overall accuracy rate of 96.49% (7,647/7,924; 95% CI, 96.08–96.87%). All four HER2 grades demonstrated high classification performance, with HER2 0 and HER2 3 + exhibiting the highest discriminative capacity. The majority of misclassifications occurred between HER2 1 + and HER2 2+, consistent with the clinical difficulty in interpreting low expression thresholds (6). ROC analysis revealed that the AUC values for HER2 0, 1+, 2+, and 3 + categories were 0.992 (95% CI, 0.989–0.995), 0.981 (95% CI, 0.976–0.986), 0.968 (95% CI, 0.960–0.975), and 0.993 (95% CI, 0.990–0.996), respectively (Figure 3A).

**Figure 3:**
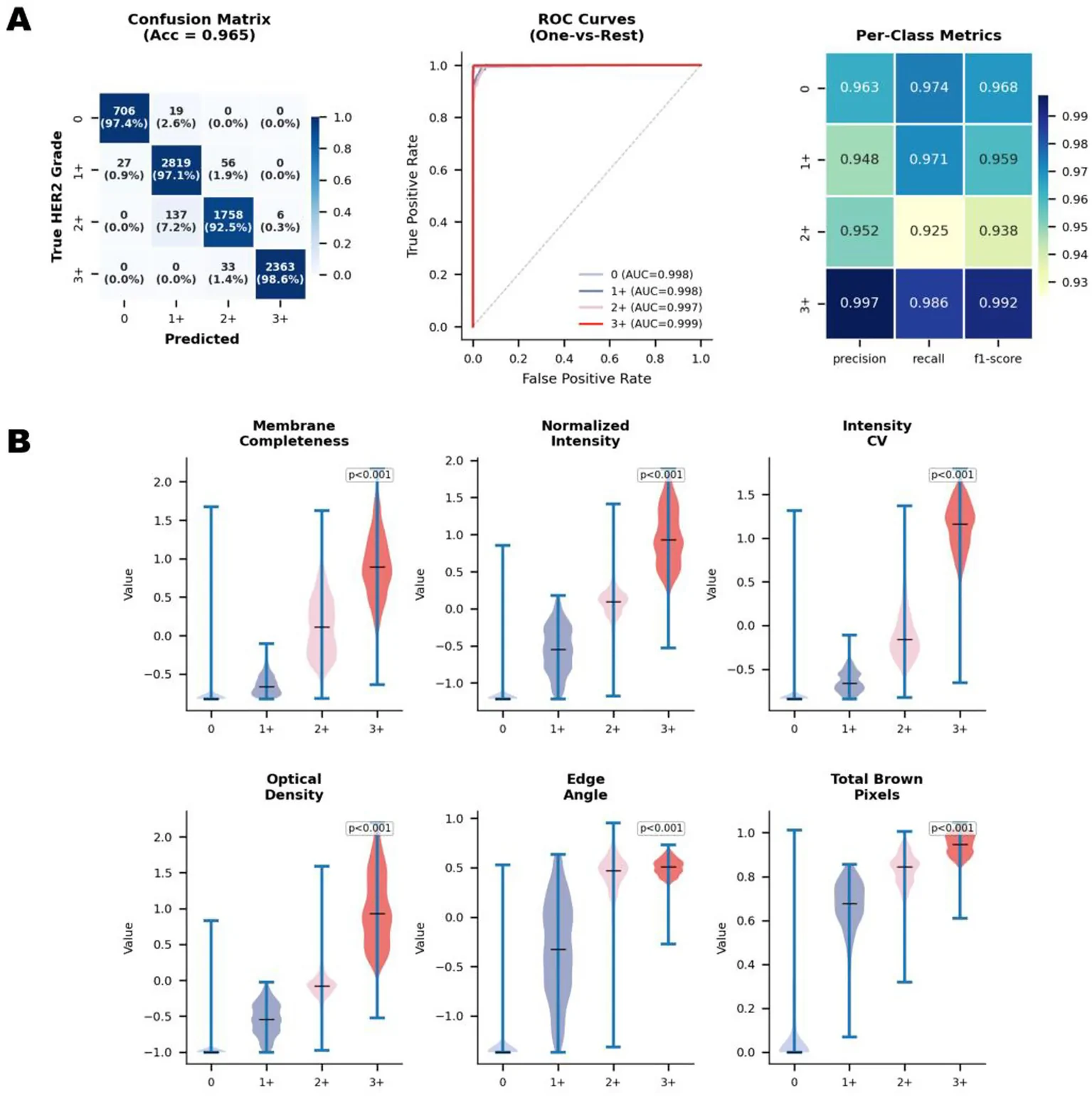
Patch-level HER2 classification performance. **(A)** Left to right: Patch-level four-class confusion matrix; one-versus-rest ROC curves for HER2 grades 0, 1+, 2+, and 3+; and per-category precision, recall, and F1-score. **(B)** Distribution of five-dimensional membrane staining features (membrane integrity, normalized intensity, coefficient of variation, optical density, and edge angle entropy) and total Brown Pixels across HER2 grades 0, 1+, 2+, and 3+. Violin plots with medians indicated by horizontal lines.

Further analysis of the membrane staining characteristics learned by the model revealed significant differences among different HER2 levels for all five cellular membrane morphological features and Total Brown Pixels (*p* < 0.001). With increasing HER2 expression, membrane integrity, normalized staining intensity, and optical density exhibited an upward trend, while the coefficient of variation for staining intensity gradually decreased, indicating that high-expression cases demonstrate more continuous and homogeneous membrane staining patterns (Figure 3B). These membrane-feature analyses were included to provide an interpretable link between the quantitative cellular representations and HER2 staining characteristics rather than to serve as an independent model-performance endpoint.

### 3.3 Analysis of cell membrane staining characteristics and topological visualization using cell-graph

To further illustrate the spatial organization of cells, Figure 4 presents Delaunay Cell Graph visualizations for four representative HER2-grade patches. From HER2 0 to HER2 3+, the overall scores of cellular nodes progressively increase from low to high values, accompanied by denser connectivity in the graph structure, resulting in a more continuous spatial topology. In HER2 3 + cases, highly scored cells exhibit distinct clustering patterns, whereas only a few scattered highly scored cells are observed in HER2 0 and HER2 1 + cases. The Cell Graph with white backgrounds further highlights the differences in spatial topologies across different HER2 grades. Across the external test cohort, the mean node degree was 5.8 ± 0.7 and the mean graph clustering coefficient was 0.42 ± 0.08. The clustering coefficient of high-scoring cells increased from 0.21 ± 0.06 in HER2 0 cases to 0.53 ± 0.09 in HER2 3 + cases, indicating progressively stronger spatial aggregation of highly expressing cells with increasing HER2 expression (see Supplementary Table S2). These quantitative observations support the use of cellular topology as a complement to membrane staining morphology.

**Figure 4:**
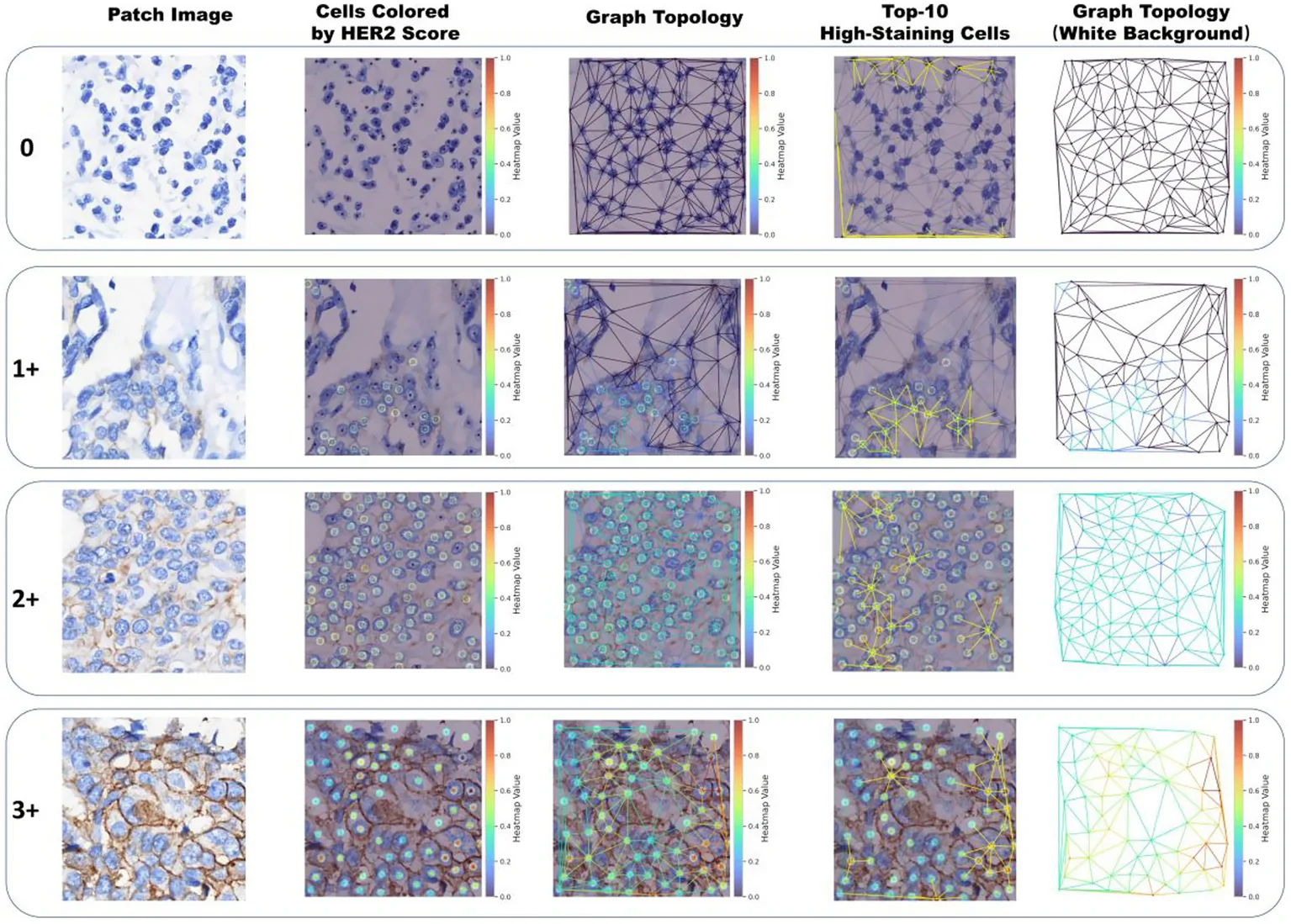
Delaunay cell-graph visualization across HER2 grades. Each row corresponds to HER2 0, 1+, 2+, and 3 + cases; columns show the original HER2 IHC image, comprehensive cellular HER2 scores, complete Delaunay Cell Graph, top-10 high-scoring cells, and Cell Graph topology (white background). Cellular scores and spatial connectivity density increase progressively with HER2 expression level.

### 3.4 Spatial analysis of invasive regions and performance of WSI-based HER2 grading

We further evaluated the model’s whole-slide classification performance across 82 independent external WSI tests.

After patch prediction fusion and spatial consistency optimization, the model achieved a WSI-level HER2 classification accuracy of 98.0% (80/82) (95% CI, 91.5–99.9%), with a quadratic-weighted Cohen’s κ of 0.97 (95% CI, 0.94–1.00). All misclassified cases occurred between HER2 1 + and 2+, with no cross-classification errors observed between HER2 0 and HER2 3+. Using the prespecified uncertainty threshold, 7 of the 82 external WSIs (8.5%) were classified as borderline uncertain and flagged for additional pathological review.

To further evaluate cross-center generalizability, complete three-center combinatorial validation was performed. Specifically, data from two institutions were used for model development, while the remaining institution was completely excluded from training and validation and used exclusively for testing. The three configurations were Changhai Hospital + 902nd Hospital → Naval Medical University, Changhai Hospital + Naval Medical University → 902nd Hospital, and 902nd Hospital + Naval Medical University → Changhai Hospital. Across the three experiments, the mean WSI-level accuracy was 96.9% ± 1.0%, the mean quadratic-weighted Cohen’s κ was 0.95 ± 0.02, and the mean patch-level accuracy was 95.8% ± 0.7% (Supplementary Table S1). These results indicate consistent performance across unseen institutional domains.

As shown in Figures 5–7, the model is capable of integrating HER2 expression data from different infiltrative regions across the entire slice scale and delineating spatial heterogeneity of HER2 distribution within tumors. Cases with low HER2 expression exhibit lower cellular HER2 scores and limited spatial clustering, whereas cases with high expression demonstrate more continuous regions with elevated scores. Ultimately, the WSI-based classification results show high consistency with pathologist assessments.

**Figure 5:**
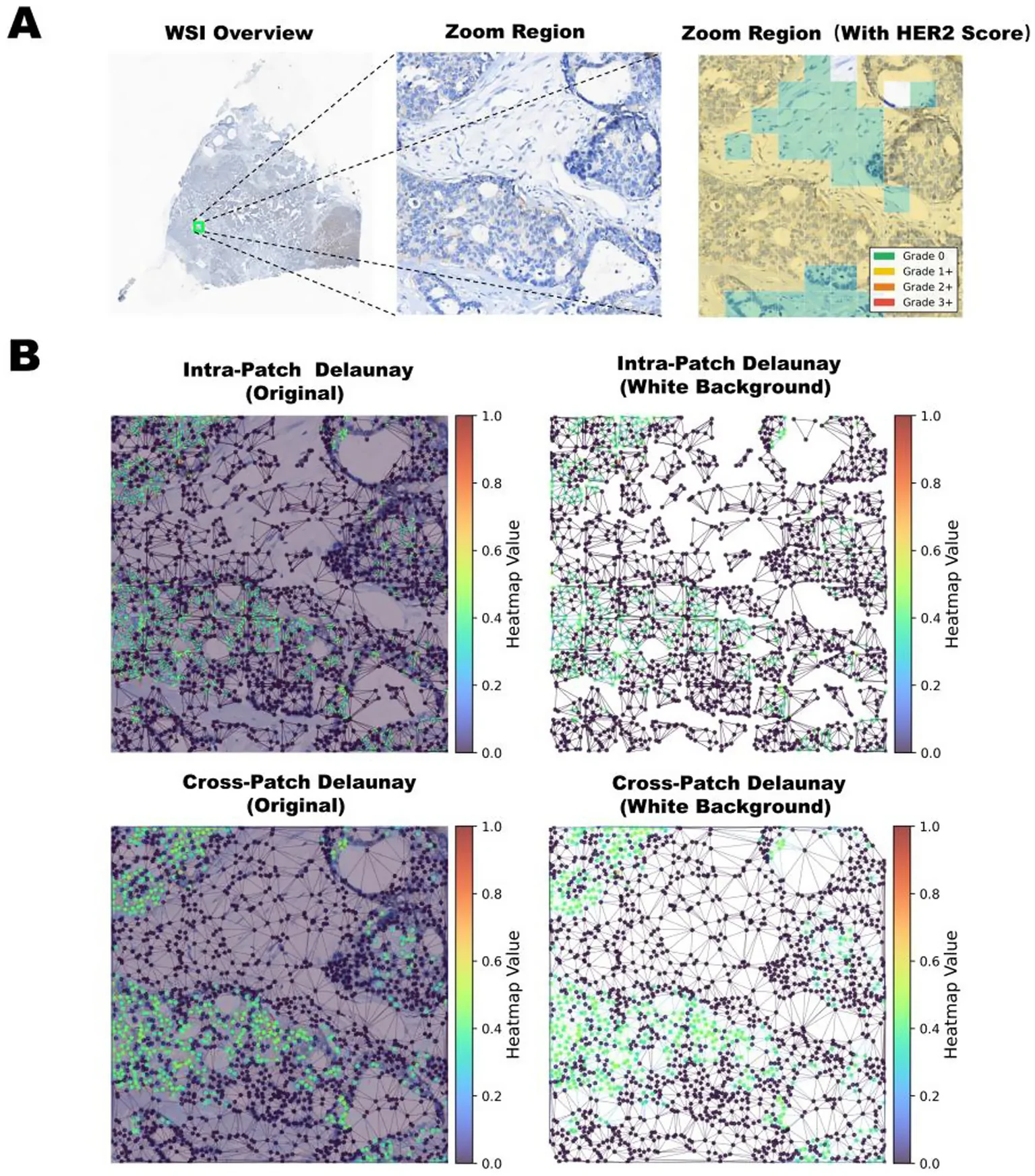
Spatial analysis of low HER2 expression cases (HER2 0/1+). **(A)** WSI overview, zoom region, and zoom region with HER2 score heatmap. **(B)** Intra-patch and cross-patch Delaunay Cell-Graph topologies, with and without IHC background; nodes and edges are color-coded by HER2 expression intensity, and the edge connectivity reflects the spatial proximity and local density of cells. Low-expression cases exhibit lower cellular HER2 scores and sparser spatial connectionsy.

**Figure 6:**
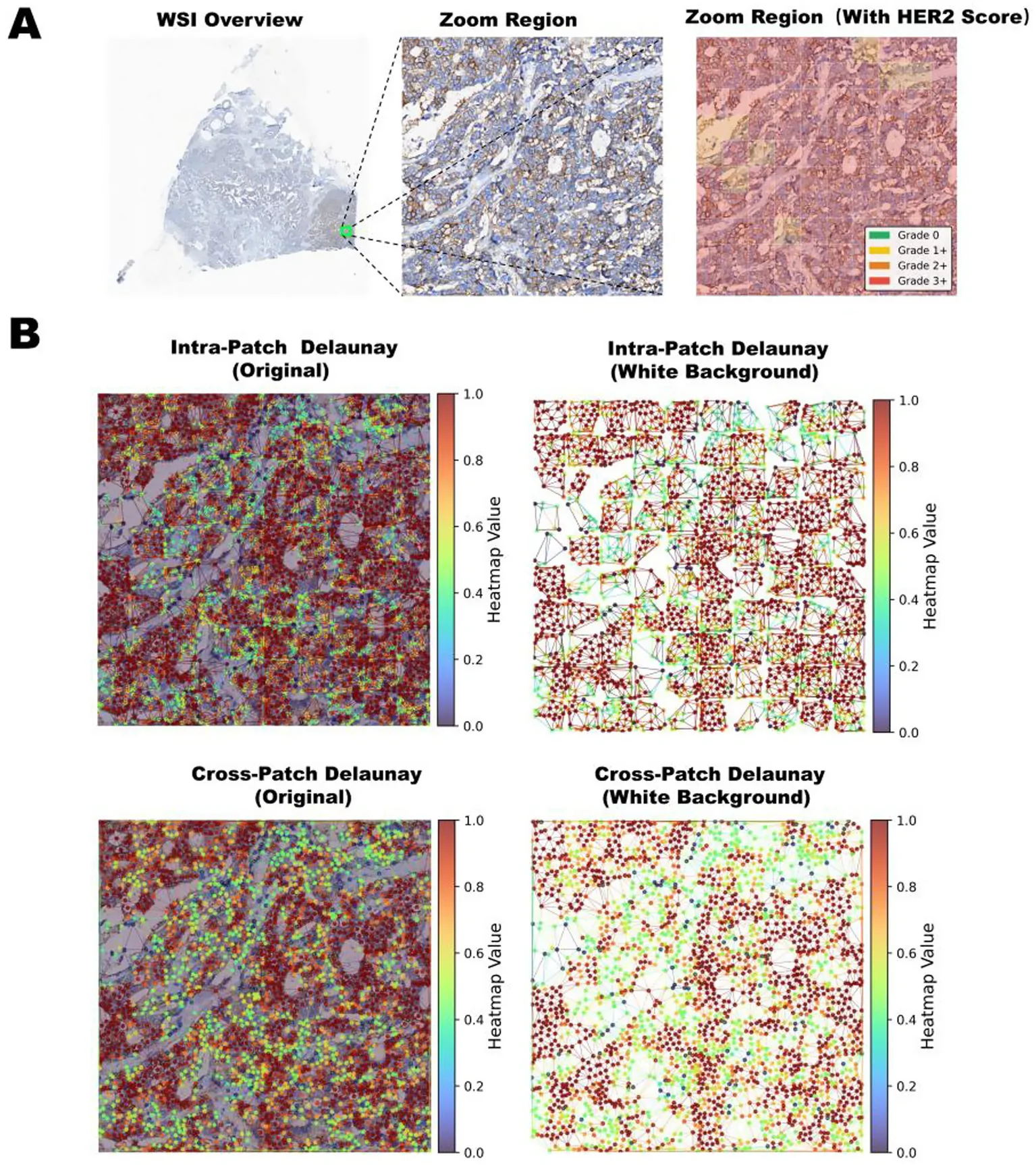
Spatial analysis of representative high HER2 expression cases (HER2 2+/3+). **(A)** WSI overview, zoom region, and zoom region with HER2 score heatmap. **(B)** Intra-patch and cross-patch Delaunay Cell-Graph topologies, with and without IHC background; nodes and edges are color-coded by HER2 expression intensity, and the edge connectivity reflects the spatial proximity and local density of cells. High-expression cases exhibit higher cellular HER2 scores, denser spatial connections, and more continuous high-expression regions.

**Figure 7:**
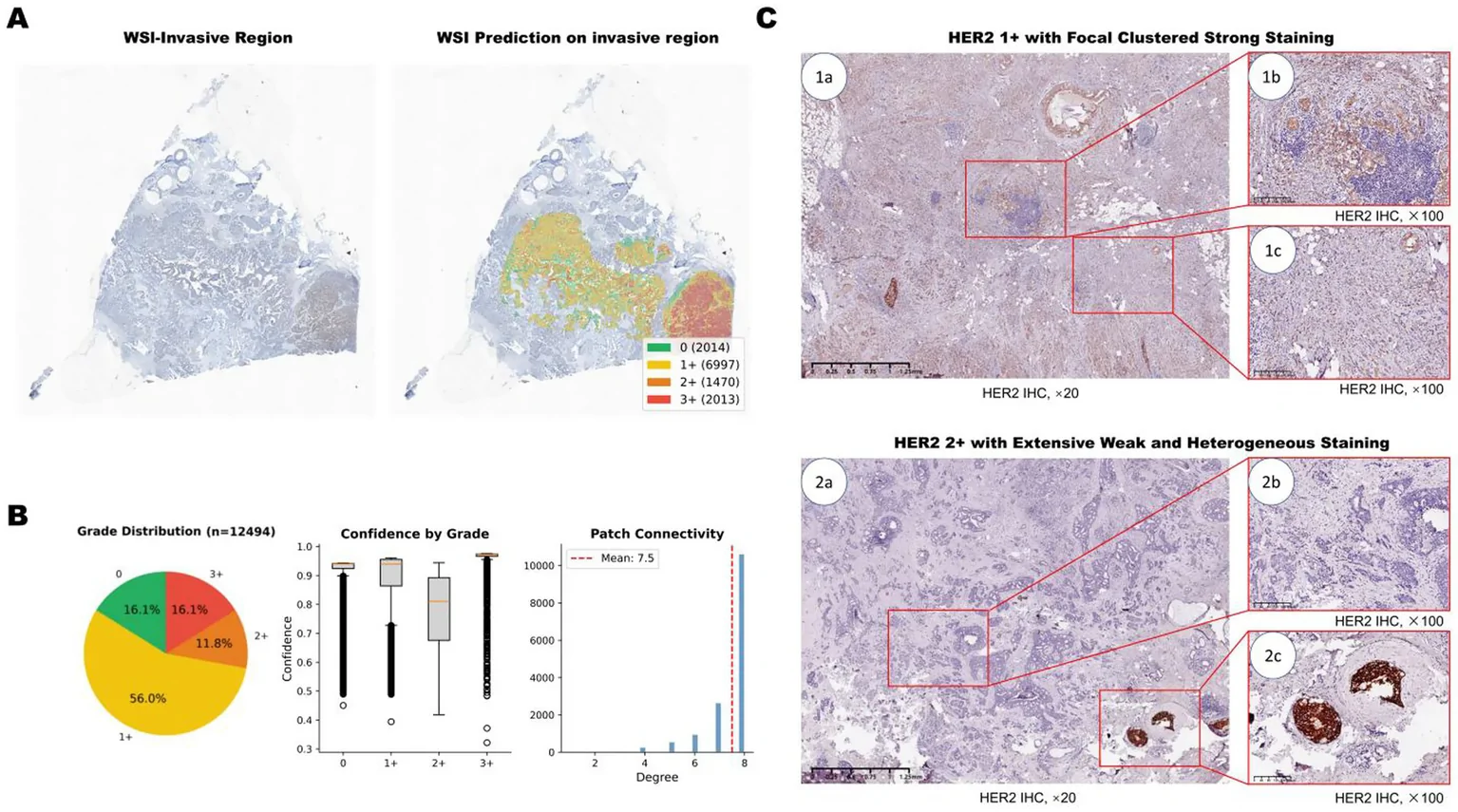
WSI-level HER2 automated grading and representative error analysis. **(A,B)** Representative analysis workflow showing invasive region detection, patch-level HER2 predictions, spatial distribution of HER2 expression, and final WSI-level scoring. **(C)** Case-level analysis of the two WSI-level misclassified cases at the HER2 1+/2 + boundary. The upper row (1a–1c) shows a HER2 1 + case misclassified as HER2 2+, including the whole-slide image (1a), a high-magnification region with focal clustered strong membrane staining (1b), and a representative HER2 1 + region (1c). The lower row (2a–2c) shows a HER2 2 + case misclassified as HER2 1+, including the whole-slide image (2a), a high-magnification region with extensive weak membrane staining (2b), and a representative HER2 2 + region (2c). IHC, immunohistochemistry.

To further investigate the two WSI-level misclassified cases, an error analysis was performed at the HER2 1+/2 + boundary (Figure 7C). The upper row (1a–1c) shows a HER2 1 + case misclassified as HER2 2+. The whole-slide image (1a) provides the overall tumor context, while the high-magnification image (1b) shows a focal region with clustered relatively strong membrane staining. In contrast, panel 1c shows a representative region with the expected HER2 1 + staining pattern. The lower row (2a–2c) shows a HER2 2 + case misclassified as HER2 1+. The whole-slide image (2a) demonstrates the overall tumor distribution, while the high-magnification image (2b) shows relatively extensive weak membrane staining. Panel 2c presents a representative region with the expected HER2 2 + staining pattern. These cases indicate that focal clustering of relatively strong staining in HER2 1 + tumors and extensive weak staining in HER2 2 + tumors may contribute to residual classification errors at the HER2 1+/2 + boundary.

### 3.5 Systematic comparison with existing HER2 automatic grading methods

To further position the proposed framework within the current landscape of automated HER2 assessment, we systematically compared it with six representative deep learning–based HER2 IHC grading methods reported in recent years (Table 4). The comparison focused on key methodological components, including invasive region localization, membrane morphology modeling, cell graph construction, WSI-level spatial modeling, and the requirement for additional immunohistochemical staining.

**Table 4:** Provides a systematic comparison with existing automated grading methods for HER2 immunohistochemistry (IHC).

Study	Invasive region localization	Membrane morphology modeling	Cell graph modeling	WSI spatial modeling	Extra staining required
Che et al. (8)	✓	✓ (handcrafted)	✗	Patch voting	✗
Xiong et al. (9)	✓	✓	✗	Sliding-window aggregation	✓ (Calponin)
Wang et al. (10)	✗	✗	✗	Transformer pooling	✗
Lu et al. (11)	✗	✗ (H&E only)	✓	Graph pooling	✗
Selcuk et al. (12)	✗	✗	✗	Multiscale aggregation	✗
Ma et al. (13)	✗	✗	✗	Patch aggregation	✗
Proposed	✓	✓	✓	Hierarchical graph learning	✗

Overall, existing approaches have demonstrated the effectiveness of convolutional neural networks, Transformer-based foundation models, and graph neural networks for automated HER2 assessment. However, most methods focus primarily on visual feature learning and perform WSI-level prediction through patch voting, probability aggregation, or global pooling. Although graph-based approaches have introduced spatial topology modeling, they are generally developed on H&E images rather than HER2 IHC slides and therefore do not explicitly incorporate HER2 membrane staining characteristics. In addition, some methods improve invasive carcinoma localization by introducing auxiliary immunohistochemical staining, which increases both experimental complexity and clinical cost.

In contrast, the proposed framework integrates automatic invasive carcinoma region detection, self-supervised representation learning, explicit HER2 membrane morphology modeling, Delaunay cell graph construction, and hierarchical graph learning into a unified pipeline. Without requiring additional staining, the framework jointly models membrane staining morphology, cellular spatial topology, and WSI-level spatial heterogeneity, providing an interpretable multi-scale solution for automated HER2 grading.

### 3.6 Ablation experiment

To evaluate the contribution of each key module to HER2 automated grading performance, ablation experiments were conducted on an independent external test set of School of Basic Medical Sciences (SBMS) (Table 5). Using CNN feature extraction combined with Patch Voting as the baseline model, we progressively incorporated DINOv2 self-supervised features, cell membrane morphological features, Cell-Graph, hierarchical graph attention networks, and a WSI-level spatial consistency optimization module.

**Table 5:** Impact of different modules on HER2 automatic grading performance (ablation experiment).

Model	Patch Accuracy (%)	WSI Accuracy (%)	Weighted κ	Description
Baseline (CNN + Simple Patch Voting)	91.2 (95% CI: 90.6–91.8)	92.7 (95% CI: 84.9–96.8)	0.89 (95% CI: 0.85–0.93)	CNN extracts patch features and uses weighted voting to obtain WSI-level results.
+ DINOv2	93.4 (95% CI: 92.9–93.9)	94.5 (95% CI: 87.4–98.2)	0.92 (95% CI: 0.88–0.95)	Introduce self-supervised pre-training.
+ 5 membrane features	94.6 (95% CI: 94.1–95.1)	95.9 (95% CI: 88.4–99.1)	0.94 (95% CI: 0.91–0.97)	Explicitly incorporating the quantitative characteristics of membrane staining.
+ Cell-Graph	95.5 (95% CI: 95.1–95.9)	96.8 (95% CI: 89.9–99.4)	0.95 (95% CI: 0.92–0.98)	Add Cell Graph Topology.
+ Hierarchical GAT	96.1 (95% CI: 95.7–96.5)	97.5 (95% CI: 91.2–99.8)	0.96 (95% CI: 0.93–0.99)	Introducing a multi-layer graph attention network.
+ Graph-cut aggregation (Complete Model)	96.5 (95% CI: 96.1–96.9)	98.0 (95% CI: 91.5–99.9)	0.97 (95% CI: 0.94–1.00)	Complete Model: Weighted Voting + WSI Spatial Consistency.

The results demonstrate progressive performance gains following the sequential incorporation of the proposed modules. With the incorporation of DINOv2 self-supervised features, patch-level accuracy improved from 91.2 to 93.4%, indicating that large-scale unsupervised tissue representation strengthens the model’s ability to capture morphological information. Further integration of five-dimensional membrane staining features raised the accuracy to 94.6%, confirming the critical supplementary role of explicit HER2 membrane expression features in deep visual representation.

By incorporating Cell-Graph and hierarchical graph attention networks, the model further enhanced its WSI-level prediction performance, with accuracy rising from 95.9 to 97.5% and a quadratic-weighted Cohen’s κ reaching 0.96, indicating that cellular spatial topology provides additional information for modeling HER2 heterogeneity at the whole-slide scale.

Ultimately, after incorporating WSI-level spatial consistency optimization, the complete model achieved optimal performance, delivering a Patch-level accuracy of 96.5%, a WSI-level accuracy of 98.0%, and a quadratic-weighted Cohen’s κ of 0.97 on an independent external test set (Table 5).

## 4 Discussion

Accurate HER2 assessment has become increasingly important in breast cancer because therapeutic decision-making has evolved beyond the traditional HER2-positive versus HER2-negative paradigm. The emergence of HER2-low breast cancer and the widespread clinical application of antibody-drug conjugates have substantially increased the demand for standardized, reproducible, and quantitative HER2 interpretation. Although digital pathology and artificial intelligence have shown considerable promise in assisting HER2 assessment, most existing methods remain limited by independent processing of invasive tumor detection and HER2 grading, insufficient modeling of spatial heterogeneity, and limited interpretability. In this study, we developed an end-to-end hierarchical graph learning framework that integrates automatic invasive carcinoma detection, cell-level membrane morphology analysis, graph-based spatial reasoning, and WSI-level prediction into a unified computational pathology workflow. The proposed framework achieved high diagnostic performance across three independent institutions and different scanner platforms, demonstrating promising robustness under the multicenter and cross-scanner conditions evaluated in this study.

One of the major strengths of our framework lies in its explicit incorporation of pathological knowledge into deep learning. Unlike conventional CNN- or Transformer-based approaches that mainly rely on image appearance, our method combines quantitative membrane staining characteristics with graph representations of cellular spatial organization. HER2 scoring in routine pathology depends not only on staining intensity but also on membrane completeness, continuity, and the spatial distribution of positive tumor cells. Therefore, explicitly modeling membrane morphology together with Delaunay cell topology enables the network to learn biologically meaningful representations that better approximate the diagnostic reasoning process of pathologists.

Another important contribution is the hierarchical modeling of tumor spatial heterogeneity at the whole-slide level. Breast cancers frequently exhibit heterogeneous HER2 expression across different tumor regions, particularly in HER2-low and equivocal cases. Conventional patch-voting strategies assume that all image patches contribute equally to the final diagnosis and therefore often ignore spatial continuity among neighboring tumor regions. In contrast, our hierarchical graph architecture propagates information between adjacent patches while preserving local tissue topology, allowing the model to capture both regional consistency and global spatial organization. This design substantially improved WSI-level robustness, as confirmed by the ablation experiments and the high agreement with expert pathologists.

The proposed framework may be particularly valuable in the era of HER2-low breast cancer. Distinguishing HER2 0 from HER2 1 + and differentiating HER2 1 + from HER2 2 + represent some of the most challenging tasks in routine pathology because these categories are characterized by weak and often heterogeneous membrane staining. While such distinctions are clinically relevant for the emerging HER2-low category, it must be emphasized that only IHC 1 + or IHC 2 + with FISH-negative results define HER2-low status (e.g., for trastuzumab deruxtecan eligibility per DESTINY-Breast trials) (4, 5). Since our model predicts IHC scores without FISH confirmation, it should be regarded as an aid for IHC interpretation rather than a direct determinant of HER2-low status or treatment eligibility. In our external validation cohort, all WSI-level misclassifications were confined to the clinically adjacent HER2 1 + and HER2 2 + categories, whereas no confusion occurred between HER2 0 and HER2 3+, suggesting that the proposed framework preserves clinically meaningful grading boundaries while maintaining high overall diagnostic accuracy.

Compared with previously published HER2 grading systems, our framework provides several methodological advantages. Che et al. extracted handcrafted membrane staining features but did not explicitly model cellular topology (8). Xiong et al. incorporated serial Calponin-stained sections to facilitate invasive carcinoma localization, although additional staining increases laboratory cost and workflow complexity (9). Transformer-based pathology foundation models have recently demonstrated excellent representation learning capability but generally lack explicit pathological interpretability. Meanwhile, graph-based computational pathology studies such as SlideGraph+ have demonstrated the importance of spatial topology but were developed using H&E rather than HER2 immunohistochemistry slides (11). By integrating automatic invasive region detection, explicit membrane morphology analysis, Delaunay cell graphs, and hierarchical graph reasoning into a unified pipeline, our method combines the advantages of these previous approaches while remaining compatible with routine pathological workflows.

Model interpretability is another important consideration for potential clinical use. Rather than functioning as a black-box classifier, the proposed framework provides multiple interpretable outputs, including invasive tumor localization, cell-level HER2 scores, graph topology visualization, spatial heatmaps, and WSI-level confidence estimation. These intermediate representations enable pathologists to visually verify the basis of automated predictions, thereby improving diagnostic transparency and potentially facilitating human–AI collaboration after prospective clinical validation. Dedicated graph-attribution approaches, such as node- or edge-level importance analysis, will be investigated in future work.

Several limitations should be acknowledged. First, although multicenter external validation was performed, all participating institutions were located in China. Additional validation using international datasets with different ethnic populations, fixation protocols, staining platforms, antibodies, and laboratory workflows is necessary before widespread clinical implementation. Second, this study employed retrospective data, and prospective clinical studies are required to evaluate the impact of AI-assisted HER2 grading on diagnostic efficiency and interobserver agreement. Third, fluorescence *in situ* hybridization (FISH) confirmation was unavailable for all equivocal HER2 2 + cases, preventing direct evaluation of AI-assisted reflex testing strategies. Finally, although the proposed framework demonstrates excellent diagnostic performance, computational efficiency and integration into digital pathology systems should be further optimized for large-scale clinical deployment. In addition, the primary reference standard in this study was expert-assigned HER2 IHC grading, and not all HER2 2 + cases had molecular confirmation by FISH; therefore, the present results should not be interpreted as direct validation of HER2 gene-amplification status.

Future studies will focus on prospective multicenter clinical validation and extension of the proposed framework to other HER2-related malignancies, including gastric cancer and colorectal cancer. Moreover, recent advances in pathology foundation models and multimodal learning provide opportunities to integrate histopathological images with genomic and clinical information, potentially enabling more comprehensive prediction of treatment response and patient prognosis. Such developments may further facilitate precision oncology and AI-assisted pathology in routine clinical practice.

## 5 Conclusion

This study established an end-to-end automated grading framework for HER2 immunohistochemistry (IHC) that integrates automatic invasive carcinoma detection, cell membrane staining analysis, and hierarchical graph reasoning. Through multicenter, cross-scanner independent validation, the framework achieved high-accuracy WSI-level HER2 scoring with a quadratic-weighted Cohen’s κ of 0.97, and all misclassifications were confined to clinically challenging 1 + versus 2 + distinctions. By jointly modeling cellular-level staining information with tumor spatial heterogeneity, this method provides a potential computational pathology tool for standardized HER2 interpretation and AI-assisted pathological assessment in breast cancer, while prospective multicenter validation remains necessary before clinical implementation.

## Data Availability

The raw data supporting the conclusions of this article will be made available by the authors, without undue reservation.
