# Supplementary material for: Automatic grading of HER2 immunohistochemical whole-slide images based on a multi-scale hierarchical graph neural network
Source: Front Public Health. 2026 Sep 29;14:1940487. doi: 10.3389/fpubh.2026.1940487 (PMC13658161; doi:10.3389/fpubh.2026.1940487)
Supplement: Supplementary file 1 [file Table_1.docx]

**Supplementary Table S1. Leave-one-center-out cross-validation results across the three participating institutions for HER2 WSI grading**

| **Training centers** | **Test center** | **WSI-level accuracy (%)** | **Patch-level accuracy (%)** | **Quadratic-weighted κ** |
| --- | --- | --- | --- | --- |
| DPCH + 902nd | SBMS | 98.0 | 96.5 | 0.97 |
| DPCH + SBMS | 902nd | 96.2 | 95.1 | 0.94 |
| 902nd + SBMS | DPCH | 96.4 | 95.8 | 0.95 |
| Mean ± SD | — | 96.9 ± 1.0 | 95.8 ± 0.7 | 0.95 ± 0.02 |
