# Supplementary material for: Automatic grading of HER2 immunohistochemical whole-slide images based on a multi-scale hierarchical graph neural network
Source: Front Public Health. 2026 Sep 29;14:1940487. doi: 10.3389/fpubh.2026.1940487 (PMC13658161; doi:10.3389/fpubh.2026.1940487)
Supplement: Supplementary file 2 [file Table_2.docx]

**Supplementary Table S2. Quantitative topological attributes of Delaunay cell graphs across HER2 IHC grades in the external test cohort.**

| **HER2 grade** | **N (WSIs)** | **Mean node degree** | **Mean clustering coefficient** | **Clustering coefficient of high-scoring cells** |
| --- | --- | --- | --- | --- |
| Overall | 82 | 5.8 ± 0.7 | 0.42 ± 0.08 | — |
| 0 | 9 | 4.8 ± 0.5 | 0.31 ± 0.06 | 0.21 ± 0.06 |
| 1+ | 28 | 5.6 ± 0.7 | 0.40 ± 0.08 | 0.34 ± 0.07 |
| 2+ | 19 | 5.7 ± 0.6 | 0.41 ± 0.07 | 0.39 ± 0.08 |
| 3+ | 26 | 6.5 ± 0.8 | 0.51 ± 0.09 | 0.53 ± 0.09 |
